# Commentary: The Glomerular Endothelium Restricts Albumin Filtration

**DOI:** 10.3389/fmed.2022.861566

**Published:** 2022-02-28

**Authors:** Wayne D. Comper

**Affiliations:** Salaqua Diagnostics Inc, New York, NY, United States

**Keywords:** charge selectivity, glomerular filtration of proteins, glomerular filtration barrier, cold isolated kidney perfusion, glycosaminoglycans

Contrary to the claims of Ballermann et al. (1) that “the glomerular endothelium bars the bulk of albumin from passing to the ultrafiltrate,” the results from the famous physiologist/thermodynamicist AG Ogston and his colleagues at Oxford would make you argue the opposite. Ogston and Preston measured the two types of albumin interaction with glycosaminoglycan chains (the major constituent of the endothelium) including equilibrium interactions reflecting excluded volume (2, 3) and transport interactions representing dynamic interactions (4) under physiological conditions. Ogston et al. found both interactions quantitatively small and unremarkable and not related to charge. When considering the extracellular partitioning of albumin from the capillary into the glomerular filtrate to be of the order of 1:0.0006 then Ogston's data would be hard pressed to account for a ratio of 1:0.4 (5).

Ballermann et al. (1) also surprisingly continue to cite work from Haraldsson's group (6–9) on the cold isolated kidney perfusion (cIPK) technique. The technique has been discredited for some time (10) particularly in relation to apparent “charge selectivity” without the criticism being addressed. The claim of glomerular charge selectivity resides in differences in the fractional clearance of albumin and that of uncharged Ficoll of the same hydrodynamic radius of 36Å. It turns out that these fractional clearance differences are near maximal at very low glomerular filtration rates (GFR) (<10% of normal). In fact, they routinely run their cIPKs at these low GFRs. Yet, when the operating GFR is increased to 50% normal the fractional clearance differences decrease by 90%. Extrapolation to normal GFR would indicate that there are no differences at all (10). The conclusion from these studies is that the apparent “charge selectivity” is massively dependent on GFR whereas genuine charge selectivity should be completely independent of it. Clearly there are other non-charge related factors determining this GFR dependence in fractional clearance (10). Other studies by this group have utilized various enzyme and chemical treatments of the kidney to affect the charge components of the endothelium and glomerular filtration barrier as a whole but the phenomenology of these studies is hardly the basis to establish a basic force in Nature.

In terms of the biophysics of albumin transglomerular transport the conclusion, from using inert transport probes that are not metabolized by kidney cells, is that charge selectivity does not exist (11–14); it is a flawed concept consistent with the original observations of Ogston et al. Therefore, while the endothelium may have a very mild effect on the size exclusion of albumin, its basic role in restricting the bulk of albumin filtration has been overstated.

## Author Contributions

WDC has been instrumental over the last 25 years in examining the biophysics transglomerular transport of proteins and delineating those concepts that are relevant and experimentally validated.

## Conflict of Interest

WDC was employed by company Salaqua Diagnostics Inc.

## Publisher's Note

All claims expressed in this article are solely those of the authors and do not necessarily represent those of their affiliated organizations, or those of the publisher, the editors and the reviewers. Any product that may be evaluated in this article, or claim that may be made by its manufacturer, is not guaranteed or endorsed by the publisher.
